# Evaluation of Commitment to Equity, Diversity, and Inclusion on Canadian Plastic Surgery Residency Program Websites

**DOI:** 10.1177/22925503261424892

**Published:** 2026-02-26

**Authors:** Meerab Majeed, Valentina Shamoun, Chantal R. Valiquette, Jana Dengler, Laura Snell

**Affiliations:** 1 Temerty Faculty of Medicine, 7938 University of Toronto, Toronto, ON, Canada; 2 Division of Plastic, Reconstructive and Aesthetic Surgery, Department of Surgery, University of Toronto, Toronto, ON, Canada; 3 Division of Plastic and Reconstructive Surgery, Tory Trauma Program, 71545 Sunnybrook Health Sciences Centre, Toronto, ON, Canada

**Keywords:** EDI, plastic surgery, residency, medical education, chirurgie plastique, EDI, enseignement de la médecine, résidence

## Abstract

**Introduction:** Equity, diversity, and inclusion (EDI) are increasingly recognized as essential to medical education. Although Canadian medical schools have advanced EDI efforts, gaps persist in postgraduate training, including plastic surgery. In the context of virtual recruitment, residency websites function as key reflections of program values. This study assesses the visibility of EDI-related content on Canadian plastic surgery residency program websites. **Methods:** We conducted a cross-sectional content analysis of all 14 plastic surgery programs participating in the 2026 CaRMS match. Program websites, CaRMS profiles, and CANPREPP pages were reviewed (August-September 2025). EDI content was assessed using 8 binary criteria adapted from Cummings et al. Two reviewers independently evaluated each site. Descriptive statistics summarized EDI scores overall and by region, language, and local visible minority population. **Results:** The mean number of EDI criteria met was 0.43 (SD = 0.76), ranging from 0 to 5. Only 1 program met more than 1 criterion. Twelve programs fulfilled zero criteria. The most common elements included standalone EDI statements (n = 2), followed by isolated mentions in mission statements, dedicated pages, or appointed leadership (n = 1 each). No programs displayed underrepresented minority-specific supports, EDI-related video content, or transgender-inclusive messaging. Stratified findings showed slightly higher scores in Anglophone programs (0.55) and cities with >30% visible minority populations (1.00), though overall representation remained limited. **Conclusion:** Canadian plastic surgery residency program websites show sparse and inconsistent EDI representation. Strengthening online EDI communication is critical to promoting equitable recruitment and diversity in surgical education.

## Résumé

**Introduction :** L'équité, la diversité et l'inclusion (EDI) sont de plus en plus considérés comme essentiels pour l'enseignement de la médecine. Même si les écoles de médecine canadiennes ont progressé en matière d'EDI, des lacunes subsistent en formation supérieure, y compris en chirurgie plastique. Dans le contexte du recrutement virtuel, les sites Web qui présentent les programmes de résidence sont une vitrine importante des valeurs. La présente étude évalue la visibilité du contenu lié à l'EDI dans les sites Web des programmes de résidence en chirurgie plastique. **Méthodologie :** Les chercheuses ont procédé à une analyse transversale du contenu des 14 programmes de chirurgie plastique qui participent au jumelage du CaRMS en 2026. Ils ont examiné les sites Web des programmes, les descriptions du CaRMS et les pages du CANPREPP en août et septembre 2025. Ils ont évalué le contenu sur l'EDI à l'aide de huit critères binaires adaptés de Cummings et coll. (2024). Deux analystes ont évalué chaque site de manière indépendante. Les statistiques descriptives ont permis de résumer les scores d'EDI globaux, de même que par région, par langue et par population des minorités visibles. **Résultats :** Le nombre moyen de critères d'EDI respectés s'élevait à 0,43 (ÉT = 0,76), selon une plage de 0 à 5. Un seul programme respectait plus d'un critère. Douze programmes n'en respectaient aucun. Les éléments les plus courants étaient les énoncés autonomes sur l'EDI (n = 2), suivis de mentions isolées dans les énoncés de mission, des pages consacrées à la question ou de leadership nommé (n = un cas chacun). Aucun programme n'affichait de soutien aux minorités sous-représentées, de vidéo liée à l'EDI ni de message sur le respect des personnes transgenres. Les résultats stratifiés ont révélé que les scores étaient légèrement plus élevés dans les programmes anglophones (0,55) et les villes composées à plus de 30 % de populations minoritaires (1,00), mais la représentation globale demeurait limitée. **Conclusion :** L'information sur l'EDI est rare et inégale dans les sites Web des programmes canadiens de résidence en chirurgie plastique. Il est essentiel de renforcer l'information sur l'EDI en ligne pour promouvoir un recrutement équitable et la diversité dans l'enseignement de la chirurgie.

## Introduction

Canada has one of the world's most ethnically and culturally diverse populations, with more than 450 backgrounds reported in the 2021 consensus.^1^ A diverse medical trainee cohort is better equipped to understand and meet the individualized needs of a heterogenous patient population, which is essential for a speciality that serves individuals across backgrounds.^2^ Increased representation underrepresented minorities (URMs) among residents and faculty fosters cultural competence, reduces implicit bias, and improves trust and communication with patients, which are critical for surgical decision-making and postoperative care.^3,4^

Equity, diversity, and inclusion (EDI) initiatives within medical training are not only ethical imperatives, but also embed practical strategies for improving the quality and accessibility of surgical care for all patients. Trainees who are from underrepresented backgrounds are more likely to serve similar underserved communities or demographics and advocate for equitable care.^5,6^ Furthermore, there is evidence that when there is greater concordance in the race, gender and ethnic background between the patient and provider, patients report greater satisfaction, improved communication and increased trust in their providers.^7,8^

Diversity remains limited across surgical residency programs, with URMs comprising a disproportionately small share of trainees relative to graduating medical student cohorts and the general population.^9–11^ This gap is even more pronounced within surgical subspecialties. Within plastic surgery specifically, the lack of diversity has been well documented, despite multiple calls to action.^12–14^ Persistent systemic barriers continue to limit opportunity, underscoring the need for visible EDI commitments and intentional recruitment strategies to foster a more inclusive surgical workforce.^15^

Applicants from underrepresented groups are more likely to select programs where they see a cultural fit, which includes sharing similar attitudes, beliefs, and values with potential colleagues.^16^ One critical way in which programs communicate their culture and commitment to diversity for potential underrepresented applicants is through the program's website.^16^ However, residency programs lack clear, consistent definitions, and criteria for URMs, and EDI content on program websites and social media is often insufficient, which can deter diverse applicants.^17^ Regarding the current landscape, the Best Practices in Applications & Selection working group noted that Canadian residency training program websites often focused on excellence in research and innovation, rather than messages about inclusivity.^18^ Additionally, a cross-sectional analysis of overarching Canadian PGME websites found that EDI representation such as mission statements, antidiscrimination policies, and mental health resources, varied considerably across programs and regions.^17^

This study is the first to evaluate the extent to which Canadian plastic surgery residency websites signal a commitment to EDI to prospective applicants. This will inform recommendations for the development of standardized EDI benchmarks across programs and regions to improve the accessibility, transparency, and inclusivity of recruitment materials.

## Methods

### Study Design

We conducted a cross-sectional, web-based content analysis of EDI information on accredited Canadian plastic surgery residency program websites during a prespecified data-collection period (August-September 2025). Fourteen programs participating in the 2026 Canadian Resident Matching System (CaRMS) match were identified. Each program's website, along with program descriptions on CaRMS and Canada's Portal for Residency Program Promotion (CANPREPP), was systematically reviewed.

### EDI Criterion Selection

The study adopted the 8 EDI criteria developed by Cummings et al,^15^ which represents the only published, plastic surgery-specific instrument to undergo psychometric evaluation and confirmatory factor analysis. Each criterion was retained in its conceptual form and operationalized as a binary variable (present = 1, absent = 0). The 8 criteria captured institutional commitment, dedicated EDI messaging, trainee recruitment supports, documented diversity initiatives, a dedicated diversity page or section, appointed diversity leadership, explicit EDI mentions in program videos, and explicit support or inclusion of transgender and gender-diverse communities (Table 1).

**Table 1.** EDI Website Criteria Adapted From Cummings et al,^15^ Used to Evaluate Program Visibility.

Criterion		Description
C1	Commitment statement in leadership message	Mentions of diversity, equity, or inclusion in the program's mission statement, program director's message, or department chair's message
C2	Standalone diversity mission statement	A separate diversity mission statement (standalone statement of the same commitment elsewhere on the site)
C3	Trainee recruitment supports for URM learners	Mentions of rotations, electives, or funded opportunities for underrepresented students (eg, Indigenous, racialized, LGBTQ2S+, low-income, rural)
C4	Active diversity initiatives	Mention of diversity initiatives (any resource dedicated to promoting diversity or inclusion within a program aside from diversity in mission statement, ie, diversity council, diversity groups, calendar, newsletter, certificate program, etc)
C5	Dedicated diversity page/section	Presence of a diversity page or section
C6	Appointed EDI leadership	Named individuals or roles (eg, EDI Officer, EDI Committee Chair) responsible for EDI efforts
C7	EDI mention in informational video	Videos or other media explicitly mention EDI values or showcase diverse trainees/faculty
C8	Explicit inclusion of transgender/gender-diverse communities	Support for transgender individuals beyond surgical services (eg, inclusive language, community support, nonbinary visibility)

Abbreviations: EDI, equity, diversity, and inclusion; URM, underrepresented minority.

### Data Collection

A point was awarded for each item identified on any of the websites reviewed. Information was considered present if it was directly available on the website, accessible through hyperlinks from departmental or affiliated promotional pages, included in embedded videos, or contained within documents related to the 2026 CaRMS cycle linked on the site. Two investigators (MM and VS) independently assessed the presence or absence of EDI elements, with discrepancies resolved by consensus. Websites presented in French were translated using Google Translate.

### Analysis

Descriptive statistics were used to summarize the number of EDI criteria met by each residency program. Mean scores and standard deviations were calculated overall and stratified by geographic region, language of study, and local population diversity. Groupings were based on provincial location, program language, and visible minority population percentages. No inferential statistics were applied due to limited sample size.

Local population diversity was estimated using data from the 2021 Canadian Census, released by Statistics Canada in October 2022.^1^ Specifically, visible minority population percentages were extracted for each residency program's corresponding census metropolitan area. Programs were stratified into 3 categories based on the proportion of racialized individuals in their region: <20%, 20% to30%, and >30%. Although recent national-level demographic data are not yet available, the 2021 Census remains as the most current and comprehensive source for ethnocultural diversity in Canada. This approach aligns with prior studies examining equity-related content in medical education websites.^19^

## Results

Fourteen Canadian plastic surgery residency program websites were evaluated using 8 predefined criteria related to EDI. Across all programs, the mean number of criteria fulfilled was 0.43 (SD = 0.76), with scores ranging from 0 to 5. The most fulfilled criterion was the presence of a standalone EDI statement (n = 2), observed on 2 program websites, followed by EDI mentions in mission statements (n = 1), dedicated EDI pages (n = 1), and appointed EDI leadership (n = 1), present in a single program website. Only 1 program met more than 1 criterion, fulfilling 5 out of 8 criteria. Twelve programs met none of the criteria. No programs demonstrated evidence of URM-specific opportunities, EDI mentions in videos, or transgender-inclusive content as seen in Table 2.

**Table 2.** EDI Elements Present on Canadian Plastic Surgery Residency Program Websites (n = 14).

EDI Criterion	Number of Programs Meeting This Criterion
C1: EDI mention in mission statement or leadership message	1
C2: Standalone diversity mission statement	2
C3: Rotations/fellowships for URM students	0
C4: Diversity initiatives (councils, newsletters, etc)	1
C5: Dedicated diversity page or section	1
C6: Appointed diversity leadership	1
C7: EDI mention in informational videos	0
C8: Support for transgender communities beyond surgical context	0

Abbreviations: EDI, equity, diversity, and inclusion; URM, underrepresented minority.

When stratified by geographic region, language of study, and local population diversity, EDI scores revealed distinct contextual patterns (Table 3). Programs located in Central Canada demonstrated the highest mean EDI score (0.63 ± 1.77), while those in Western and Eastern Canada averaged 0.20 and 0.00, respectively. Anglophone programs scored higher on average (0.55 ± 1.42) than Francophone programs. Stratification by local population diversity, using city-level census data, showed that programs situated in cities with >30% visible minority populations had the highest mean EDI score (1.00 ± 2.83), followed by those in the 20% to 30% range (0.17 ± 0.38), while programs in cities with <20% minority populations scored 0.00 across the board.

**Table 3.** Equity, Diversity, and Inclusion (EDI) Elements Present on Canadian Plastic Surgery Residency Program Websites (n = 14) Stratified by Geographic Region, Language of Study, and Regional Population Diversity.

Program Characteristic	Mean EDI Score	SD (±)
Geographic region		
Western Canada (n = 5)	0.20	0.40
Central Canada (n = 8)	0.63	1.77
Eastern Canada (n = 1)	0.00	0.00
Language of study		
Anglophone (n = 11)	0.54	1.42
Francophone (n = 3)	0.00	0.00
Local population diversity		
<20% minority (n = 4)	0.00	0.00
20%-30% minority (n = 5)	0.17	0.38
>30% minority (n = 5)	1.00	2.83

## Discussion

This study is the first to examine EDI content on Canadian plastic surgery residency program websites. Overall, representation was minimal, with most programs meeting none of the predefined criteria and only isolated examples of commitment statements. While modest variation was observed by region, language, and local population diversity, the overarching finding is a striking absence of EDI visibility across the specialty. These findings suggest that geographic and linguistic context may influence the visibility of EDI commitments, but do not guarantee their presence, underscoring a broader systemic gap in program-level engagement with equity and inclusion. This reflects the slow rate of diversification of plastic and reconstructive surgery compared to other specialties.

Our findings show Canadian plastic surgery residency programs average just 0.43 EDI criteria per website, with 86% scoring zero. In contrast, US programs evaluated by Cummings et al^15^ averaged 2.1 criteria using a comparable framework. Other US plastic surgery studies—He et al^16^ (mean 3.4 elements), Das et al^20^ (48% with ≥1 element), and Choi et al^21^(average 3.1 elements)—report higher EDI visibility but rely on broader or differing criteria, including visual representation and social media activity. Within the Canadian context, plastic surgery programs also ranked lower compared to other specialties: ophthalmology programs averaged 2.4 EDI elements, dermatology programs averaged 6.8 out of 29 elements (with 92% meeting at least 1), and a national review of all Canadian PGME programs found an average of 8.65 out of 20 EDI criteria, with over half meeting at least 10.^17,19,22^ Regardless of scoring framework, Canadian plastic surgery programs consistently demonstrate the lowest EDI visibility across both specialty and institutional benchmarks.

The absence of visible EDI content on Canadian plastic surgery residency program websites may have significant implications for equity in recruitment, particularly for applicants from underrepresented backgrounds. Institutional diversity has been shown to influence residency program selection among applicants from underrepresented backgrounds.^23–25^ As such, the inclusion of EDI-related content on program websites may serve as a meaningful signal of inclusion, as many applicants rely on website messaging to evaluate programs.^26^ Across specialties, programs that feature explicit EDI messaging, dedicated diversity leadership, and targeted support for URM applicants consistently attract more diverse applicant pools and demonstrate improved representation among trainees.^27–29^ Within the Canadian context, there have been calls for the integration of EDI principles into recruitment, education, and leadership in plastic surgery, emphasizing that visible allyship and inclusive messaging are essential to fostering a representative and culturally competent surgical community.^12^

The limited EDI visibility observed across Canadian plastic surgery residency websites may reflect deeper structural barriers within the specialty. Prior research has highlighted the scarcity of URM faculty and leadership in plastic surgery, particularly within the academic context.^13,30^ This can hinder prioritization of EDI initiatives and limit opportunities for mentorship and visible role modelling. Without diverse leadership or dedicated EDI officers, programs may lack the institutional capacity or accountability to embed equity into recruitment methods, such as website messaging.

### Limitations

This study faced several limitations. Firstly, this study relied on information publicly available on the Canadian plastic surgery program websites and therefore may not fully capture the breadth of EDI efforts occurring through other channels such as social media platforms, medical school events, newsletters or internal documents and discourse. This may be underestimating EDI efforts happening within programs not reflected in the program websites. Additionally, for French-language program websites, reliance on direct and automated Google Translation may have limited the accuracy and nuance of content interpretation, potentially impacting the comprehensiveness of data extracted for analysis. Program size was not examined in this study and may represent an unmeasured factor influencing website quality, as larger programs may have greater financial and administrative resources to support website development and maintenance. Finally, some plastic surgery residency programs maintained standalone websites, whereas others were shorter subpages within the representing university or postgraduate medical education website. In such cases, only the subpage specific to the plastic surgery program was evaluated for criteria, which may have further undermined the EDI efforts happening at large within that university.

### Recommendations

To effectively recruit, attract, and retain diverse plastic surgery trainees, programs must demonstrate a clear and sustained commitment to EDI. This begins with making EDI commitments explicit, visible and easily accessible to prospective applicants. Programs should recognize residency websites as not only an informational platform, but as a critical equity and recruitment tool that reflects a program's culture and priorities.^31^ Evidence shows that most residency applicants rely on online resources for information about programs, with official websites serving as the most frequently used source.^32^ Programs are encouraged to align with published best practices that emphasize visible leadership engagement, measurable diversity goals, and the integration of EDI principles into recruitment materials. Drawing on Bondok et al 's^19^ framework for Canadian ophthalmology programs, plastic surgery programs should consider adopting a similarly structured approach focused on 4 key domains: (1) *showcasing team diversity*, (2) *clear discourse in recruitment*, (3) *inclusive resources*, and (4) *program organization*. Additionally, the validated criteria developed by Cummings et al^15^ offer a practical framework for assessing EDI visibility on program websites. Incorporating the recommendations can help programs identify gaps, monitor progress, and benchmark improvement across institutions. Consistent with the national call to action by Avery et al^33^ programs are encouraged to integrate EDI principles across both education and recruitment, including dedicated curricular content (eg, equity-focused morbidity and mortality rounds, journal-club discussions, and seminar materials), faculty and trainee bias training, and defined goals for inclusive excellence within the CaRMS process.

## Conclusion

Canadian plastic surgery residency program websites demonstrate the lowest EDI visibility among both national and international comparators. Despite growing expectations for transparency and equity in medical education and training, most programs lack basic online indicators of EDI commitment. Addressing this gap requires intentional, visible, and systemic efforts to ensure that recruitment materials reflect the values of EDI and representation essential to the future of surgical training.
